# Tailoring silicon for dew water harvesting panels

**DOI:** 10.1016/j.isci.2021.102814

**Published:** 2021-07-01

**Authors:** Xiaoyi Liu, Joachim Trosseille, Anne Mongruel, Frédéric Marty, Philippe Basset, Justine Laurent, Laurent Royon, Tianhong Cui, Daniel Beysens, Tarik Bourouina

**Affiliations:** 1 ESYCOM Lab, UMR 9007 CNRS, Univ Gustave Eiffel, 77454 Marne-la-Vallée, France; 2 Fondation de l'Ecole Normale Supérieure, Paris, 75005, France; 3 Physique et Mécanique des Milieux Hétérogènes, CNRS, ESPCI, PSL Research University, Sorbonne Université, Sorbonne Paris Cité, 75005 Paris, France; 4 Laboratoire Interdisciplinaire des Energies de Demain, Université Paris Diderot, CNRS, Sorbonne Paris Cité, 75013 Paris, France; 5 Department of Mechanical Engineering, University of Minnesota, Minneapolis, 55455, USA; 6 OPUR, 75016 Paris, France

**Keywords:** materials design, materials property, metamaterials, surface

## Abstract

Dew water, mostly ignored until now, can provide clean freshwater resources, just by extracting the atmospheric vapor available in surrounding air. Inspired by silicon-based solar panels, the vapor can be harvested by a concept of water condensing panels. Efficient water harvesting requires not only a considerable yield but also a timely water removal from the surface since the very beginning of condensation to avoid the huge evaporation losses. This translates into strict surface properties, which are difficult to simultaneously realize. Herein, we study various functionalized silicon surfaces, including the so-called Black Silicon, which supports two droplet motion modes—out-of-plane jumping and in-plane sweeping, due to its unique surface morphology, synergistically leading to a pioneering combination of above two required characteristics. According to silicon material's scalability, the proposed silicon-based water panels would benefit from existing infrastructures toward dual functions of energy harvesting in daytime and water harvesting in nighttime.

## Highlights

• Unusual condensation dynamics occur on the hierarchical Black Silicon metasurface
• Near-zero water removal time and good water yield are simultaneously achieved
• Great scalability and multifunctionality are accessible to the proposed water panel

## Graphical abstract

**Figure:**
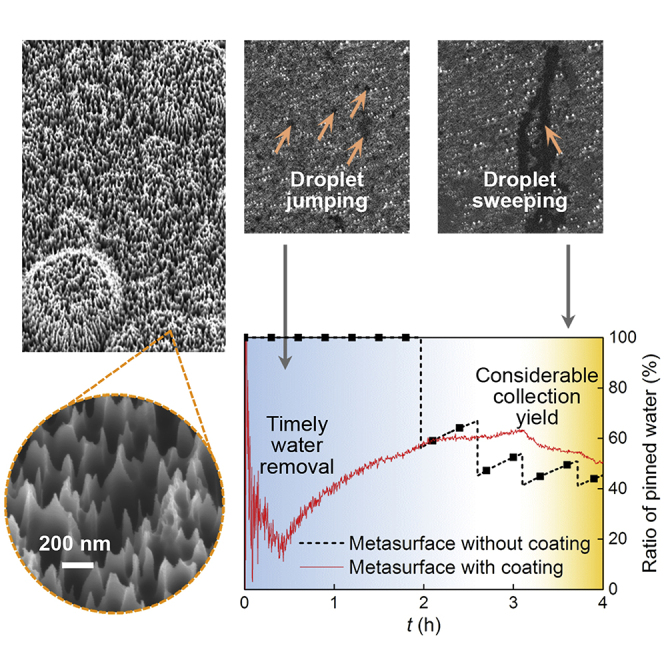

## Introduction

In the context of global warming and decrease of pure water resources, condensing atmospheric vapor is a particularly appealing solution to find new sources of pure water owing to not only the huge amount of water (12,900 km^3^) contained in atmosphere but also because this scheme has less restrictions of geographical and hydrologic conditions (Beysens, 2018). A promising approach is the use of passive radiative cooling materials, which enables substrates to lose heat by radiation and be cooled within a few degrees to generate the condensate (Beysens, 2018). Such approach requires unique optical performance such as high infrared emissivity (Zhai et al., 2017; Raman et al., 2014; Rephaeli et al., 2013). However, when dealing with water harvesting, besides the excellent optical radiative properties, additional surface functions of the material are also desirable, including very early start of water removal and very efficient water collection. The former ensures that water can be collected before re-evaporating and also leave available surface for re-nucleation and further condensation of droplets; the later determines the yield of collected water, which directly determines the performance of a condenser. These ideal behaviors are yet unattainable on common surfaces due to several limitations on their intrinsic surface properties.

In nature, a number of plants and insects have developed ingenious strategies to fulfill specific goals such as self-cleaning properties (Lotus leaves) (Barthlott and Neinhuis, 1997) and water collection abilities (Namib desert beetles) (Parker and Lawrence, 2001). These properties have in common the creation of high-quality sliding surfaces, which usually exhibit great droplet self-removal ability and can partially satisfy the requirements of effective water harvesting above (Wei et al., 2014; Hou et al., 2020). However, the water removal on such surfaces is generally driven by gravity against pinning forces, usually resulting in an unignorable waiting time and considerable evaporation loss (Beysens, 2018). Another issue is that the expected wetting properties heavily rely on specific surface morphologies. No matter biomimetic structures (Mele et al., 2012; Barthlott et al., 2010; Schulte et al., 2011; Yi et al., 2019; Kuru et al., 2011; Gao et al., 2005), anisotropic sliding surfaces (Kang et al., 2013; Wu et al., 2011; Zhang et al., 2014), or hierarchical architectures (Yan et al., 2020; Chen et al., 2007; Rykaczewski et al., 2013), they require relatively complicated fabrication steps involving lithography or chemical synthesis, which is challenging for large-area production and seriously restricts their applications.

Recently, a silicon-based needle-shaped metasurface has been developed with the popularly known name of “Black Silicon” (Sarkar et al., 2019; Oh et al., 2012; Steglich et al., 2014; Dorrer and Rühe, 2008). This material exhibits both unusual optical (Sarkar et al., 2019; Oh et al., 2012; Steglich et al., 2014) and wetting properties (Dorrer and Rühe, 2008) due to its sub-micrometer scale roughness. Those two remarkable properties make it a very good candidate both for radiative cooling as well as water harvesting. Herein, we study a specific type of Black Silicon covered by a 40 nm-thickness polytetrafluorethylene (PTFE) film, which can act as a truly sliding surface with a large contact angle (CA) and minute contact angle hysteresis (CAH). It is shown to support both the droplet out-of-plane jumping motion mode and in-plane sweeping mode, accordingly achieving a pioneering combination between the near-zero preparation time of water removal and the considerable water harvesting yield which rarely coexist in prior reports (Qu et al., 2015). Essentially, the dynamic CA induced by the spontaneous-formed hierarchical surface (Saab et al., 2014; Nguyen et al., 2013) (see below in Discussion) plays a key role in this process. Other unusual condensation dynamics characteristics of the proposed metasurface related to various important functions, such as heat transfer and dew harvesting (Beysens, 2018; Bintein et al., 2019; Gerasopoulos et al., 2018; Trosseille et al., 2019; Lv et al., 2015; Boreyko and Chen, 2009), are also investigated. Significantly, the processing of such metasurfaces can be completed in one single equipment within tens of minutes, which may provide enormous advantages in mass production and subsequent integration toward multifunctional platforms, especially considering the compatibility and scalability of silicon material. Details are given below.

## Results

**Figure 1:**
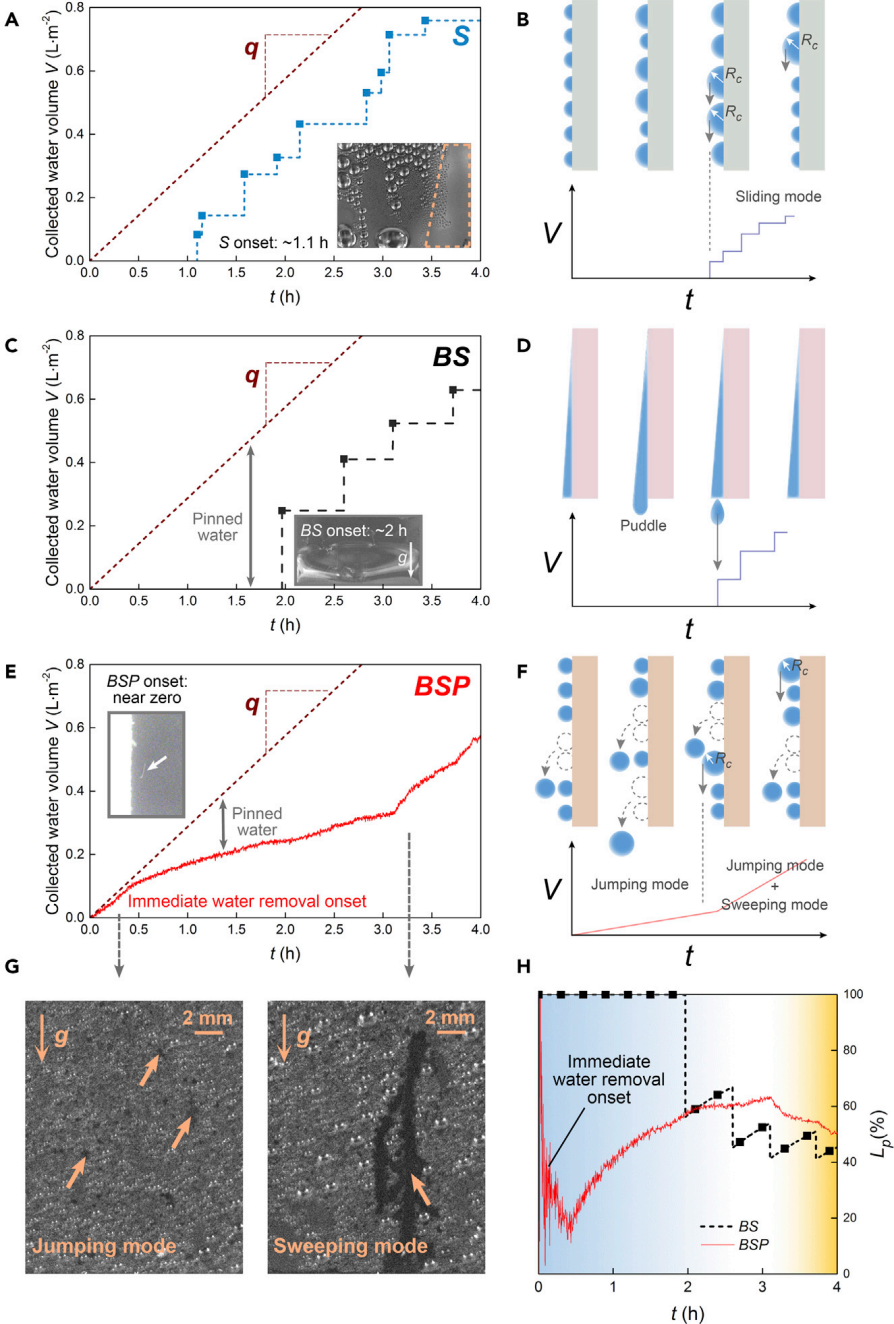
Efficient water harvesting based on hierarchical architecture (A), (C) and (E) Evolution of the collected water volume (*V)* per unit area of *S*, *BS,* and *BSP*. The dotted line is the total condensed water volume versus *t*. The gap between total condensed water volume and collected water volume is the pinned water volume on sample surface. The insets respectively present the first shedding events of *S*, *BS,* and *BSP*. (B), (D), and (E) Diagrams of water removal from *S*, *BS,* and *BSP*. *S* and *BS* present stepwise curves of water collection, while *BSP* presents a distinct continuous rising curve. (G) Remained traces of droplet jumping and sweeping departure modes. The former corresponds to the scattered dots, while the latter corresponds to a branching pattern. (H) The ratio of pinned water volume *L*_*p*_ versus *t* of *BS* and *BSP*. The blue area is the interval which *BSP* performs advantages compared with *BS*. The orange area is the interval which the water collection efficiency of *BSP* accelerates obviously.

We fabricated 2 samples of Black Silicon without/with PTFE film, indicated as *BS* and *BSP*, and plus a sample of silicon (called *S*), to serve as a reference. The CA and CAH measurements illustrate that the wetting properties of Black Silicon have been totally shifted from superhydrophilic to superhydrophobic by a few nm thick layer of PTFE (see Figure S1, the CA and CAH of *S* sample are also given). First, we evaluated the water harvesting abilities of the three above samples in a climatic chamber (see METHOD DETAILS and Figure S2). In this case, samples are set vertically in the chamber to obtain the volume of collected water. Figure 1A, Figures 1C and 1E, respectively, represent the collected water volume (*V*) falling from *S*, *BS* and *BSP* samples versus time (*t*)*.* Figures 1B, 1D, and 1F give their corresponding schematics of water removal characteristics*.* The total volume of condensate versus *t* is also given (see METHOD DETAILS and Figure S3), while its slope is the condensation rate *q*. For *S*, each rising step on the curve denotes a droplet shedding event at the critical size *R*_*c*_. For *BS*, where filmwise condensation occurs, a puddle forms at the bottom of the substrate and each rising step on the curve denotes an event of puddle detachment. Detachment occurs when the puddle section area becomes on order the square of the capillary length *l*_*c*_ (Lee et al., 2012). For both *S* and *BS*, the number of shedding events is clear and discernible. The insets in Figures 1A and 1C are photos recording the first shedding events on *S* and *BS*, which respectively present typical dropwise and filmwise condensation based on ordinary and superhydrophilic surfaces, as well as relatively late water removal onset. Compared with *S* and *BS*, the *BSP* sample presents a surprisingly continuous rising curve instead of the stepwise curve. It signifies that on such sample, frequent but small, even indistinguishable droplet shedding events lead to the rising of *V*. In this case, the water removal is driven by an out-of-plane droplet departure mode, namely, jumping behavior, which is the consequence of droplet coalescence between two or more droplets, only appearing on ultra-low adhesion surfaces (Boreyko and Chen, 2009; Miljkovic et al., 2013; Tian et al., 2014). More importantly, in the first few minutes of condensation, the jumping behavior has already started, leading to a near-zero water removal onset time of *BSP* (see the inset in Figure 1E). This near-zero onset, to our knowledge, has never been reported in previous literatures (Beysens, 2018; Bintein et al., 2019; Gerasopoulos et al., 2018; Trosseille et al., 2019) and is a key feature for efficient water harvesting against evaporation of pinned water on surfaces.

In addition to the jumping behavior, another droplet departure mode, the sweeping behavior, which is induced by the droplet in-plane coalescence and occurs later than jumping, also exists on *BSP*. The droplet in sweeping motion will merge and take away the neighboring droplets through a series of coalescence along its motion track, leading to remarkable surface refreshing and water removal abilities (Qu et al., 2015; Dai et al., 2018). Therefore, sweeping has been confirmed as an excellent approach for water collection. Figure 1G presents the photos of distinct remained traces left by droplet departure through jumping and sweeping motion. The ultimate yield of collected water from *BSP* is quite considerable, which also results from the combination of droplet jumping and sweeping modes, as shown in Figure 1F.

To illustrate more instinctively the effective water collection efficiency of *BS* and *BSP*, we define *L*_*p*_ as the ratio of pinned water volume to the total condensate volume, where the amount of pinned water signifies the probable evaporation loss induced by untimely collection. It is reflected by the gap between *V* and the total condensate volume (tilt dotted line) shown in Figures 1C, 1E. 1H presents the *L*_*p*_ of *BS* and *BSP* versus *t*. Due to the late water removal onset time, the *L*_*p*_ of *BS* remains 100% until ~2 hr; on the contrary, the *L*_*p*_ of *BSP* decreases rapidly since the very beginning of condensation owing to the droplet jumping behavior, even achieves to ~20%. The water collection efficiency of *BSP* is always higher than that of *BS* until ~2.5 hr, which means that for short-term water harvesting (blue area in Figure 1H), the *BSP* is a much better choice since huge evaporation loss can be avoided by the timely water collection. On the other hand, hydrophilic surfaces are usually implemented to harvest water from atmosphere owing to the advantages of drop nucleation (Parker and Lawrence, 2001; Dai et al., 2018); however, attributed to the sweeping behavior, the water collection efficiency of hydrophobic *BSP* accelerates obviously in the late condensation (yellow area in Figure 1H), resulting in an ultimate yield like that of hydrophilic *BS*. The co-existing of droplet jumping and sweeping motion on *BSP* accordingly enables the pioneering combination between early water removal and considerable water harvesting yield to be attainable.

As the reference, a brief comparison of several representative water harvesting studies was made, as shown in Table 1. In the case of environmental and cooling conditions close to this work, the *BSP* metasurface exhibits good water harvesting capacities, especially in the late stage of condensation. On the other hand, the near-zero water removal onset time demonstrated by *BSP* is also rarely reported in relevant studies. Details of relative humidity (RH) and subcooling settings of present experiment can be found in the METHOD DETAILS.

**Table 1:** Comparison of several representative studies on the topic of Dew Water Harvesting

Literature	Surface materials and configuration	Moisture provided by	RH (%)	Subcooling (°C)	Water harvesting capacity (L⋅m^−2^⋅h^−1^)	Water collection onset time
Bintein et al. (2019)	Epoxy/SiO_2_ grooves	Climatic chamber	–	–	~0.2	0.4–1.3 hr
Jin et al. (2017)	Polymer flat surfaces	Humidifier	100	17	~0.2	0.28–0.72 hr
Hou et al., 2020	Patterned copper	Humidifier	~50	24	~1.1	–
Gerasopoulos et al. (2018)	Nanostructured Teflon AF & PTFE surfaces	Chamber	98	27	~0.72	0.17–0.25 hr
Xu et al. (2020)	Porous coating layer	–	~95	3–5	~0.13	–
Trosseille et al. (2019)	Rough Duralumin alloy plates	Climatic chamber	70	6.5	~0.15	0.7–1.4 hr
Our work	Needle-shaped Black Silicon	Climatic chamber	70	10	Early stage: ~0.1 Late stage: ~0.27	Near-zero

The subcooling indicates the temperature difference between ambient air and the surface where water condensation occurs.

## Discussion

**Figure 2:**
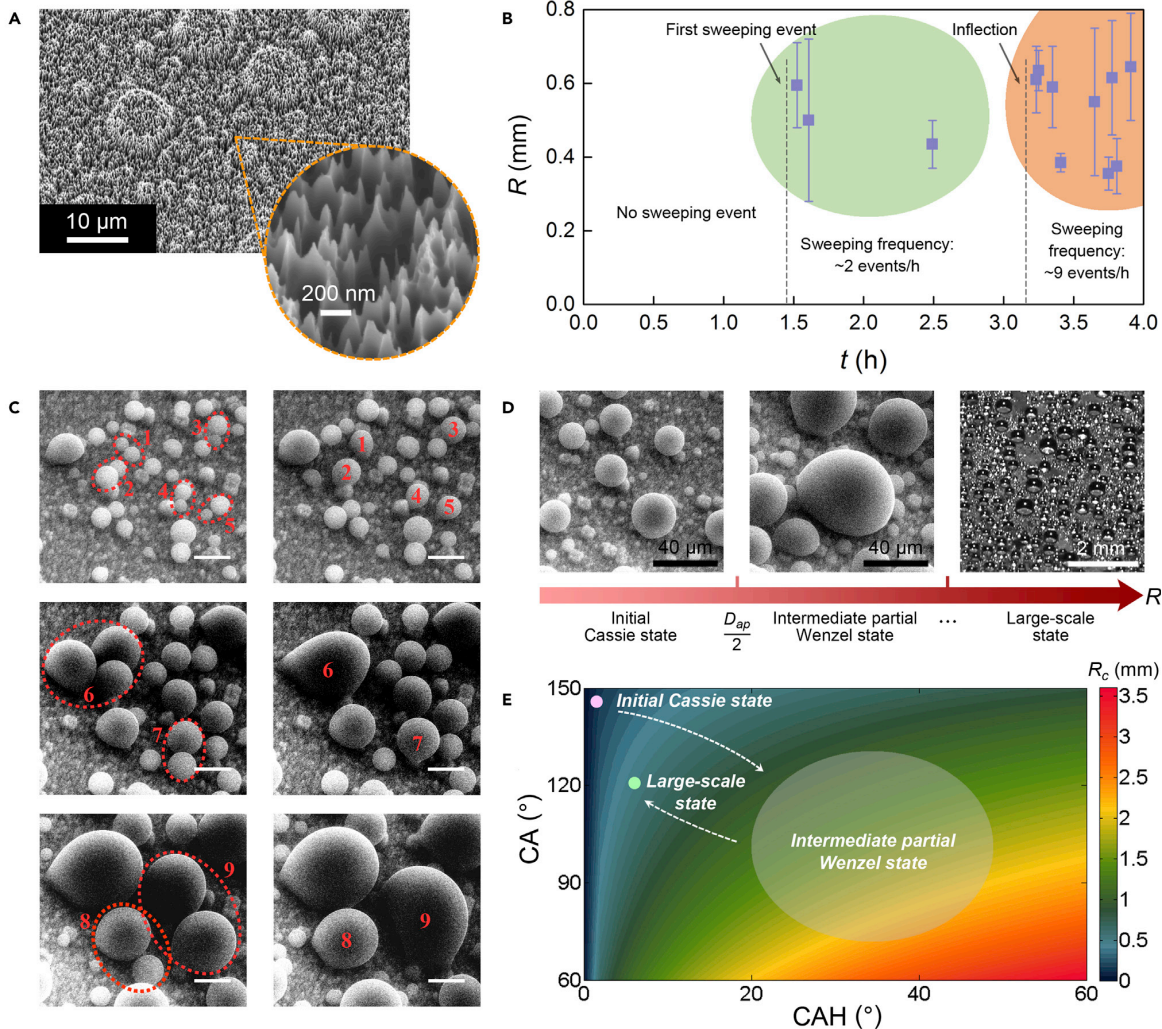
Dynamic CA based on the hierarchical surface (A) SEM photos of hierarchical *BSP* surface. (B) The occurrence time and droplet shedding radius in each sweeping event. The error bar signifies the calculated radius range according to each main trace. The sweeping frequency is not high in the green interval, but much higher in orange interval. (C) Three couples of ESEM photos for presenting the coalescence-induced conversion from initial Cassie-Baxter state to intermediate partial Wenzel state. The scale bars are 20 μm. (D) Diagram of three condensation phases on *BSP*. The first two photos are taken in the same area for the sample piece, with a time interval of 48 s. (E) Actual critical radius *R*_*c*_ in sweeping behavior calculated by the dynamic CA and CAH for *BSP*. Different colors represent different calculated values of *R*_*c*_. The diagram of droplet evolution tracks from phase (i) to phase (iii) is also given, illustrating the evolution trend of actual *R*_*c*_.

According to prior studies, the coexistence of jumping and sweeping behaviors requires rigorous conditions (Qu et al., 2015; Chu et al., 2016), which often occurs on the surfaces with complicated nanostructures (Wen et al., 2017; Mohammadian et al., 2020; Peng et al., 2019; Mukherjee et al., 2019). In jumping, the motion direction of resulting droplet is perpendicular to the substrate (Yan et al., 2019), while the sweeping motion, which requires an in-plane velocity, will be easily prevented in this case (Qu et al., 2015). In order to understand the rare droplet motion herein, we firstly observe the unique surface morphology of *BSP*. Figure 2A shows the scanning electron microscopy (SEM) photos of *BSP* surface covered by PTFE thin film, while a reference photo of *BS* is also given (see Figure S4). It is an obvious hierarchical architecture comprising micro-patches and nano-needles with high aspect ratio, which is spontaneously formed during the etching process (Saab et al., 2014; Nguyen et al., 2013). Such substrate will thus exhibit different wetting properties and consequent water motion modes compared with simple sliding surfaces.

Figure 2B presents the time of each sweeping event on vertical *BSP*, as well as the radius of shedding droplet calculated by the corresponding trace. The error bar signifies the calculated radius range according to each main trace (see Figure S5). It can be seen that the first sweeping event occurs at ~1.5 hr, and the sweeping frequency is not high before ~3 hr (green area in Figure 2B); however, the sweeping events occurs much frequently after ~3 hr (orange area in Figure 2B), resulting in the acceleration of water collection efficiency in Figure 1F. Notably, the sweeping events generally occur with a relatively stable critical droplet radius of ~0.5 mm, which is too small to cause obvious steps on the water collection curve, consequently leading to the continuous rising one (by observing the droplets shedding from *S*, the formation of typical stepwise curve requires the shedding radii of at least ~3 mm). According to the directions of sweeping events, gravity can be considered as the dominant way to drive the motion.

If simplifying the sweeping as the sliding, the critical radius *R*_*c*_ can be calculated by the balance condition between pinning and gravity forces acting on droplet along the surface plane (Quéré et al., 1998; ElSherbini and Jacobi, 2006):

(Equation 1) $$k^{*}\gamma \left(\cos\;\theta_{r}-\cos\;\theta_{a}\right)R\;\sin\;\theta_{c}=\frac{\text{π}}{3}R^{3}\left(2-3\;\cos\;\theta_{c}+{\cos\;}^{3}\theta_{c}\right)\rho g\;\sin\;\alpha$$

where *k∗* = 48/π^3^ is a parameter depending on the droplet contour shape; *R* is the radius of droplet; *ρ* and *γ* are density and surface tension of water; *α* = 90° is the angle between sample surface and horizontal plane; *θ*_*r*_ = 147.9° (ranges from 146.7° to 150.7° in measurements) and *θ*_*a*_ = 148.4° (ranges from 147.2° to 149.6° in measurements) are respectively advancing CA and receding CA of *BSP*, which were measured in advance (see Method details and Figure S1). Uncertainties in CA gives a CAH = 1.45° ± 1.45°; *θ*_*c*_ is the static CA of *BSP*, which can be approximated as the average value of *θ*_*r*_ and *θ*_*a*_ since the CAH of *BSP* is quite small. Then *R*_*c*_ can be theoretically calculated (Beysens, 2018; Trosseille et al., 2019; ElSherbini and Jacobi, 2006). When Δ*θ* (namely, CAH) < 1.45°, *R*_*c*_ is approximately less than 0.14 mm, which is far away from the observed results of ~0.5 mm. The reasons for this discrepancy are discussed hereafter.

Then we observe the droplet condensation state on *BSP* by environmental scanning electron microscope (ESEM) to explore the mechanism of sweeping. Figure 2C presents three couples of ESEM photos for neighboring droplets coalescence. We can see that the coalescence will break the initial perfect Cassie-Baxter state of small droplets and turn the resulting droplets into intermediate partial Wenzel state. This situation is due to the hierarchical biphilic surface topography of *BSP*, consisting of conical structures with high aspect ratio leading to Cassie-Baxter state and hydrophilic patches with low aspect ratio nanostructure leading to Wenzel state (see SEM photos of Figure 2A). The growth of condensed droplets on such surface overall exhibits three phases: (i) majority of drops nucleate on the top of hydrophilic patches and independently maintain an initial large CA; (ii) then a metastable Cassie-Baxter state gradually shifts to partial Wenzel state, and the droplets are fixed on the top of patches even when merging with neighboring ones, leading to the decreased CA of the resulting droplets (Chen et al., 2011). If the distance between adjacent patches *D*_*ap*_ is relatively large, the resulting droplets will probably form a “bridge” between adjacent patches (Chen et al., 2011); in our case, they exhibit intermediate partial Wenzel state with decreased CA due to the relatively small *D*_*ap*_, which mainly concentrates in the range of ~5–30 μm, corresponding to a coalescing droplet radius $D_{ap}/2$; (iii) as the droplets continue to grow, they eventually turn into a large-scale state where they keep approximately spherical until they feel gravity. Figure 2D presents the diagram for the above three phases, including corresponding photos taken by ESEM and CCD camera.

Accordingly, the droplet critical radius *R*_*c*_ in sweeping is actually a dynamic function of CA and CAH for the condensed droplets which keep varying during condensation, as shown in Figure 2E. From phase (i) to phase (ii), the *R*_*c*_ becomes larger; on the contrary, it becomes smaller when the condensed droplets evolving from phase (ii) to phase (iii). It can explain the differences of calculated and observed results of *R*_*c*_: the former was conducted under the premise of “static CA”, while our CA and CAH are “dynamic” during the condensation. The dynamic CA is also the basis of the two water removal modes co-existence—the dominant droplet removal mode is determined by the varying CA in different phases. During condensation, the sweeping acts as a supplement of jumping to enhance the water harvesting capacity, while the jumping compensates the absence of sweeping in early condensation.

**Figure 3:**
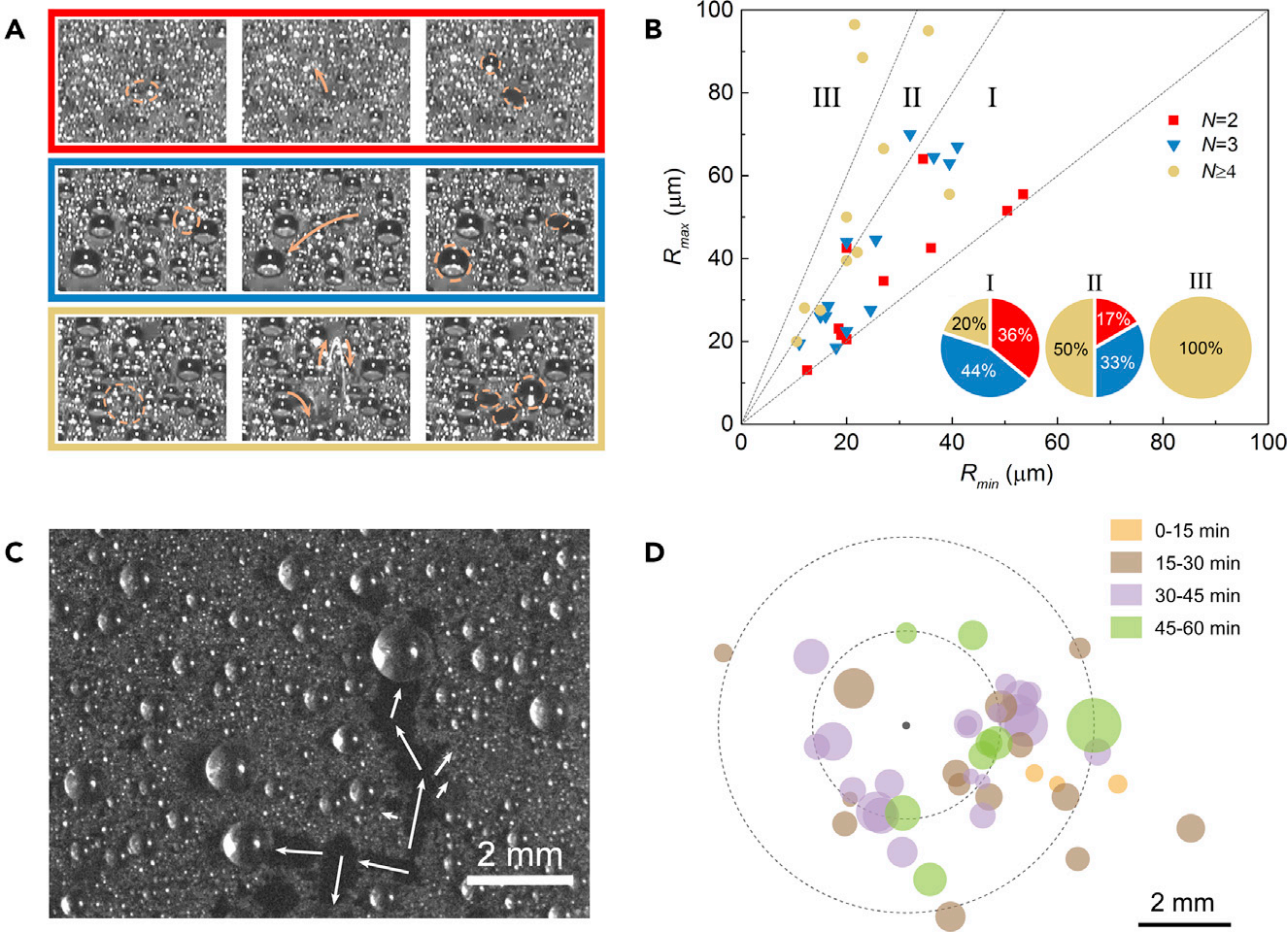
Characteristics of jumping and sweeping behaviors on horizontal *BSP* (A) Typical droplet jumping events involving droplet number *N* = 2 (red), 3 (blue) and 4 or more droplets (brown). Dotted lines mark the coalescence-involved and ultimate resulting droplets, while arrow symbols show the jumping directions. The interval between neighboring frames is 33 ms. (B) Statistical count of largest $(R_{max}$) and smallest $(R_{min}$) droplet radii involved in each observed jumping events. Different marks correspond different involved droplet number *N*. The slopes of the three dotted lines from left to right are 3, 2, and 1, respectively. The inset count the proportions of jumping events with different *N* in region Ⅰ, Ⅱ, and Ⅲ, respectively. (C) Typical remained traces of sweeping. The direction of motion is random after eliminating the influence of gravity, accompanying with the neighboring droplets coalescence along the track. (D) Diagram of direction and ultimate droplet size for each sweeping event. The circle size and its distance to origin represents the ultimately resulting droplet size and the displacement of sweeping motion, respectively. The different colors of circles signify their different occurrence time.

To further analyze the droplet jumping and sweeping behaviors, we set the *BSP* sample horizontally and observe the condensation process carefully. It can be observed that the jumping behavior indeed starts from the early beginning period of condensation (see Figure S6). Typical coalescence and consequent jumping events concerning N=2, N=3, and N≥4 drops are given in Figure 3A (also see Videos S1, S2, and S3, Typical jumping event on horizontal *BSP* with *N* = 2, *N* = 3, and *N* ≥ 4, related to Figure 3A), where *N* is the number of droplets involved in coalescence. The radii of the biggest and smallest droplets involved in each coalescence-induced jumping event are named as$\;R_{max}\;$and $R_{min}$. Figure 3B exhibits the distribution of $R_{max}$ and $R_{min}$ distinguished by different merging droplets number N. Three dotted lines with slopes of 3, 2 and 1 from left to right divide Figure 3B into three regions Ⅲ, Ⅱ, Ⅰ. Droplets involved in coalescence with significantly different sizes have difficulties to jump since the smaller droplet does not bring sufficient momentum to enable them to detach from the surface (Yan et al., 2019; Mouterde et al., 2017). The largest ratio of $R_{max}/R_{min}$ herein is ~4.55, signifying a volume asymmetry of approximate 100 times, close to the upper limit in prior reports (Yan et al., 2019; Mouterde et al., 2017). Such high mobility is achieved by virtue of multi-droplets coalescence, which is able to make it easier to overcome the energy barrier due to pinning forces and consequently leads to the promoted jumping behavior (Lv et al., 2015). As a support, the proportions of jumping events with different N in each region is reported in Figure 3B inset. It is worth mentioning that the photos in brown frame in Figure 3A not only show multi-droplets jump events but continuous double-step jumps. Such domino-like behaviors generate tangentially flying droplets which promote further coalescences (Rykaczewski et al., 2013).

Video S1. Typical jumping event on horizontal BSP with N = 2, related to Figure 3A (AVI)

Video S2. Typical jumping event on horizontal BSP with N = 3, related to Figure 3A (AVI)

Video S3. Typical jumping event on horizontal BSP with N ≥ 4, related to Figure 3A (AVI)

Different from the jumping motion, the sweeping behavior results from the in-plane droplets coalescence (Qu et al., 2015; Marcos-Martin et al., 1995). Figure 3C shows a photo of typical droplet sweeping trace on horizontal *BSP*. Clearly, the direction of sweeping motion is random after eliminating the influence of gravity, accompanying with the neighboring droplets coalescence along the motion track. We also counted the direction, the ultimate droplet size, and the occurrence time for each sweeping event, as shown in Figure 3D. The maximum ratio of motion displacement to ultimate droplet radius among all the events is up to 24.6, exhibiting the strong water removal and surface refreshing capabilities. Meanwhile, droplet sweeping behavior occurs rarely in early condensation, since the dynamic CA is not suitable for sweeping motion yet as mentioned before.

**Figure 4:**
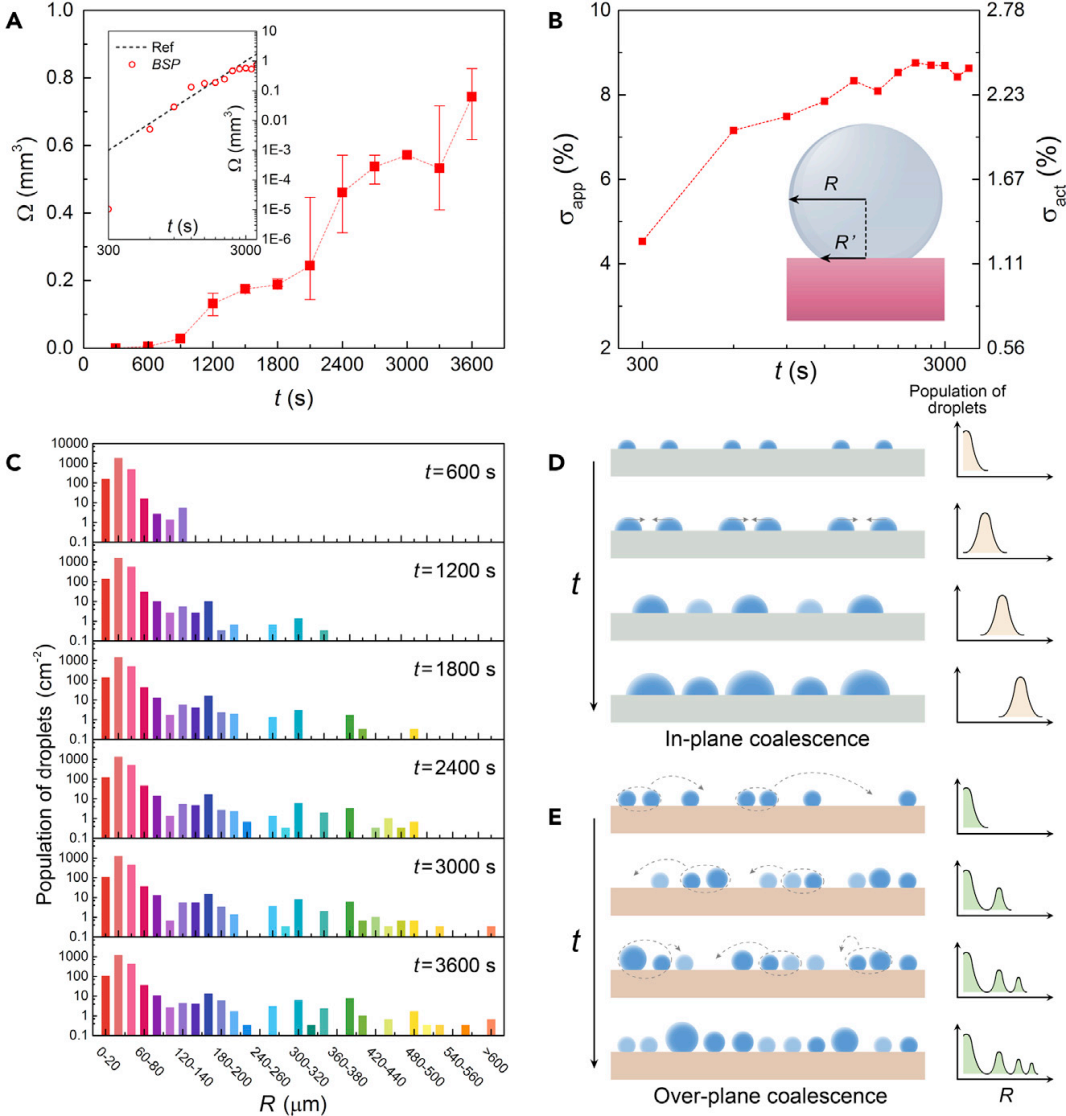
Unusual condensation dynamics on horizontal *BSP* (A) Average volume of the largest five droplets Ω on *BSP* versus time *t*. The error bars are the range among measured droplets areas. The inset is the same data displayed in logarithmic coordinates, and the dotted line in inset is a reference line with a slope of 3. (B) Evolution of the apparent surface coverage σ_*app*_ and the actual surface coverage σ_*act*_ on *BSP* surface. The inset explains the difference between σ_*app*_ and σ_*act*_. (C) Distribution histogram of droplet population versus *R* in each period. The condensation evolution on *BSP* exhibits droplet-renewal characteristics. (D) Diagram of droplet self-similar evolution trend on ordinary surface. (E) Diagram of droplet self-renewal evolution trend on *BSP* surface. Light blue droplets in (D) and (E) indicate the new generation in each frame during condensation.

As a result of both jumping and sweeping behaviors, the condensation dynamics on horizontal *BSP* performs some unusual characteristics. Firstly, the growth trend of droplets on *BSP* sample is different from that of ordinary surfaces. To reveal the growth law of the largest droplets which are mainly affected by jumping, the average volume of the largest five droplets is defined as Ω. Figure 4A represents the variation trend of Ω versus *t*, and is also drawn in logarithmic coordinates, as shown in the inset. The dotted line in inset is a reference curve with the slope of 3. Generally, droplet volume should theoretically be linear with respect to $t^{3}$ (dotted line) since the droplet radius is proportional to *t* on ordinary surfaces (Beysens, 2018). However, the droplet growth trend on *BSP* is leaping-type rather than linear one, since the largest droplets are mainly obtained through the jumping behavior which induces multi-drop coalescences.

Additionally, the droplets jumping and sweeping behaviors can leave large area of fresh space, leading to the low surface coverage shown in Figure 4B. Through image recognition, the surface coverage can be quantitatively calculated (see Figure S7). Note that, due to the large CA of *BSP*, the radius of the droplet contact line (*R′*) on *BSP* is quite different from the radius of droplet (*R*), as shown in the inset of Figure 4B. Therefore, the calculation of surface coverage on *BSP* should be considered through two approaches: the apparent coverage ($\sigma_{app}$) calculated from *R* and the actual coverage ($\sigma_{act}$) calculated from *R′*, which are related by $\sigma_{act}=\sigma_{app}\;sin^{2}\theta_{c}$. Figure 4B presents the surface coverage of *BSP* with double *y* axes of $\sigma_{app}$ and $\sigma_{act}$. The actual surface coverage on *BSP* sample is thus extremely low (lower than 7%). Even if we consider $\sigma_{app}$, the calculated ~25% surface coverage is one of the remarkable properties for hierarchical surfaces (Chen et al., 2011), even though considering the uncertainties induced by the limited image quality.

Another interesting phenomenon is the unique droplet population distribution versus size. Figure 4C presents several time-resolved histograms of droplet population versus radius *R*. The video of dynamic histograms is also given (see Video S4, Dynamic distribution histogram of droplet population versus *R* in each period with a time interval of 300 s, related to Figure 4C). It shows that the distribution of small droplets keeps differentiating into several groups where some large droplets suddenly appear instead of continuously evolving. This is the result of jumping droplets which eventually merge with other droplets when landing back on the *BSP* surface, as well as the sweeping droplets resulting from a series of coalescences along the motion track. Meanwhile, the smallest droplets continuously nucleate and grow in the space left free by jumping and sweeping drops during the whole condensation period, keeping the small droplet population relatively stable. Generally, the jumping-sweeping-induced droplet-renewal evolution mode on *BSP* surface is different from what is currently observed with ordinary surfaces, which usually exhibit a common droplets evolution mode mainly driven by in-plane coalescence of immobile droplets (Beysens, 2018). In order to intuitively explain their distinctions, drawn in Figures 4D and 4E are the diagrams of droplet evolution trends on ordinary and *BSP* surfaces, respectively. Condensation photos of each period taken from horizontal *BSP* surface are also given as reference (see Figure S8).

Video S4. Dynamic distribution histogram of droplet population versus R in each period with a time interval of 300 s, related to Figure 4C (AVI)

## Conclusions

From the perspective of practical applications in future, the utilization of proposed metasurface in combination either with grooves (Bintein et al., 2019) or with surfaces holding cascading effect (Ang et al., 2019) may lead to optimum water collection performance and get closer to the ultimate limit of “zero pinned water”. Considering the remarkable optical emissivities of Black Silicon extending to the infrared in atmospheric window waveband (Sarkar et al., 2019), a 1 m^2^ such metasurface panel can predictably provide more than 1 L of water per night in a passive manner with zero energy consumption, which is quite considerable for whether drinking or irrigation demands, and is significant to the regions with freshwater scarcity or water pollution issues. Note that the emissivity of water in atmospheric window is ~0.98, which therefore enables the radiative cooling performance of proposed panel to be still maintained when covered by the condensate. Normally, a 5–10°C anticipation of the cooling effect for an open radiative cooling system with good thermal insulation is accessible (Zhao et al., 2019). Therefore, the subcooling of *BSP* achieved by Peltier cooler in this work is reasonably expected to be implemented by radiative cooling in practice. More importantly, the total processing time of proposed metasurface merely requires 10 min in one equipment, which would be highly beneficial to mass production. Owing to the above advantages of processing, as well as the excellent wetting and optical properties, the proposed silicon-based metasurface exhibits great potential for designing large-area panel-like platforms such as high-efficiency water harvesting panels based on radiative cooling, or solar panel with self-cleaning capabilities, or even dual functions of water/energy harvesting in night/daytime.

### Limitations of the study

Although the proposed Black Silicon metasurfaces are expected to possess good radiative cooling capacities due to their remarkable optical properties, the appropriate surface modifications are required, so as to inhibit the absorption beyond atmospheric window. The feasible modification schemes should be further investigated. Meanwhile, the practical radiative cooling effect of the black silicon in the water-scarce areas should also be verified, since the actual environmental conditions all over the globe are various, which would be different from those in the experiments.

## STAR★Methods

### Key resources table

**Table:** 

REAGENT or RESOURCE	SOURCE	IDENTIFIER
**Chemicals**		

Crystalline silicon wafers (on-demand custom-made)	Si-Mat (Silicon Materials) company	CZ SS <100>, 100 mm diameter, Thickness: 525 ± 25 μm, resistivity 1–20 Ω⋅cm.

**Software**		

MATLAB	The MathWorks, Inc	MATLAB version 2019

**Other**		

Deep Reactive Ion Etching (DRIE) Micromachining etch system	Alcatel Vacuum Technology	Alcatel 601 E
Peltier Cooler	HB Electronic Components	TEC1-12706
Climatic Chamber	Weiss-Technik	Weiss WKL 100
Camera	TheImagingSource	4 MPix CCD Camera
DROP SHAPE ANALYZER	Krüss	DSA25

### Resource availability

#### Lead contact

Further information and requests should be directed to the lead contact, Dr. Tarik Bourouina (tarik.bourouina@esiee.fr).

#### Materials availability

This study did not generate new unique reagents.

#### Data and code availability

This study did not generate new code. All data and analytical methods are available in the main text.

### Method details

#### Sample fabrication

Black Silicon is produced on 4-inch single-side polished [100]-oriented silicon wafers with a resistivity of 1–20 Ω⋅cm. The process is carried out in a A601E Alcatel etch tool configured for cryogenic silicon etch at a temperature of −110°C during 10 min. The Black Silicon formation process involves an inductively coupled plasma (ICP) source power of 1000 W and a gas mixture of SF_6_ and O_2_ at a pressure of 2.5 Pa, with detailed formation mechanisms described in our previous work (Saab et al., 2014; Nguyen et al., 2013). By proper control of SF_6_/O_2_ ratio, the process leads to the appearance of conical spikes on the silicon surface. Then further control of the etching time enables the production of high aspect ratios for these conical structures. For the sample that requires further coating to make it superhydrophobic, the corresponding deposition step is done within the same plasma reactor at 0°C with C_4_F_8_ as feed gas for 10 s, leading to a 40 nm-thick fluorocarboned film, commonly assimilated to PTFE. The resulting wafers were subsequently diced into 3 cm × 2 cm dies using a dicing saw.

#### Experiments in climatic chamber

As shown in Figure S2, samples are set on a Peltier cooler to control their surface temperature. The angle between the cooler plane and horizontal is adjustable. A series of photos are taken by CCD cameras with high resolution (20 μm) from various perspectives to record the condensation or jumping events. A tissue near the substrate (within approximately 0.2 mm) is stretched to collect the shedding droplets whose mass is continuously recorded by a precision scale. The data corresponding to the collected water volume *V* from 2 samples have been treated by MATLAB to eliminate the influence of evaporation from damp tissue. Chamber adjustments are set to 20°C for the temperature of Peltier cooler surface, 30°C for air temperature and 70% for RH in Figure 1 experiments and to 0.5°C for cooler surface temperature, 20°C for air temperature and 50% for RH in Figure 3 experiments. In the first case, the RH in chamber generally went from an initial value (30%) to 70% within 15 min and subsequently become stable; as for the second case, the RH in chamber can be regarded as stable from the beginning since the ambient RH can be controlled at 50% in advance.

#### Condensation rate calculation

The condensation rate *q* was calculated through a reference experiment in the climatic chamber with the same conditions of above experiment. We took a series of photos of condensation on a tape surface which its CA is known, and the profiles of condensed droplets can be recognized by MATLAB (Trosseille et al., 2019), as shown in Figure S3a. Accordingly, the condensed water volume and consequent equivalent water film thickness of unit area can be calculated. Figure S3b shows the calculated equivalent water film thickness at different time during condensation, and the dotted line represents the total volume of condensed water versus *t*, which is obtained by fitting these data points. Herein, the slop of the dotted line is namely the condensation rate.

#### CA measurements

The CA of two samples is measured by a DSA25–Drop Shape Analyzer. Samples are fixed on a horizontal stage, and a syringe hung above them gently places a small droplet (~10 μL) on their surfaces by a precise electric pump. The drop is imaged by a CCD camera and its profile is further analyzed by software. The advanced and receding CA are measured by injecting or withdrawing water in or from the droplet, which accordingly grow or shrink. All the CA measurements are performed at ambient temperature.

#### Condensation observation in ESEM

The micro-scale period of condensation on *BSP* is observed in a Quattro Thermofisher ESEM. The sample piece is stuck on a holder by carbon tape with a tilt angle of ~45°. In experiment, the ESEM chamber is firstly evacuated and then filled by vapor with a stable pressure of 2000 Pa. Then the holder and sample are cooled by a Peltier cooler, which shifts the temperature from 22°C to 10°C by a cooling rate of 4°C/min after experiment start. Meanwhile, the RH in chamber is increased from 75% to 100% within 2 min. The working distance between imaging detector and sample piece is set as 6.7 mm. Furthermore, the electron beam voltage and current are respectively set as 28 keV and 75 pA to simultaneously guarantee the good image quality and beam heating effect inhibition.
