# Supplementary material for: Tailoring silicon for dew water harvesting panels
Source: iScience. 2021 Jul 1;24(7):102814. doi: 10.1016/j.isci.2021.102814 (PMC8319802; doi:10.1016/j.isci.2021.102814)
Supplement: Document S1. Figures S1–S8 [file mmc1.pdf]

iScience, Volume *24*

Supplemental information

Tailoring silicon for dew water harvesting panels

Xiaoyi Liu, Joachim Trosseille, Anne Mongruel, Frédéric Marty, Philippe Basset, Justine Laurent, Laurent Royon, Tianhong Cui, Daniel Beysens, and Tarik Bourouina

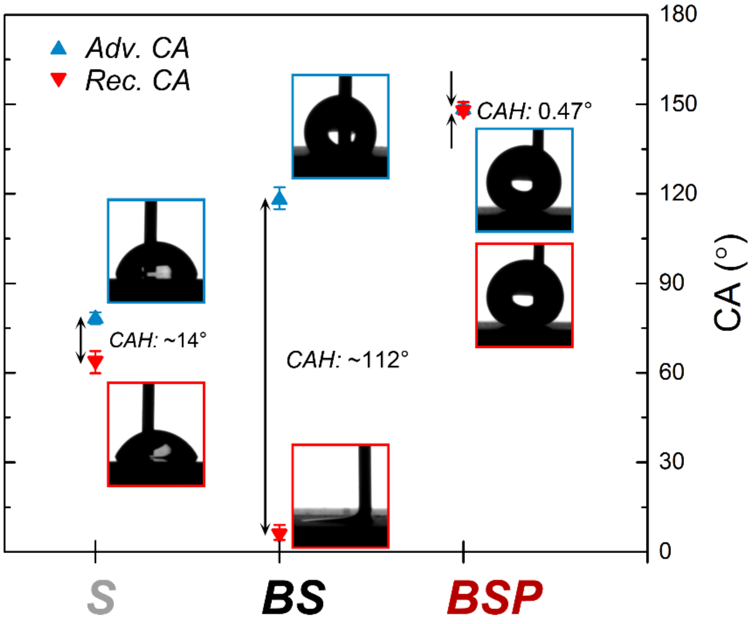

Figure S1. Advancing and receding CA measurements of *S*, *BS* and *BSP*, Related to Figure 1-4. The error bars represent the range of measurements. The superhydrophilicity of *BS* and superhydrophobicity of *BSP* are clearly demonstrated by their CA and CAH.

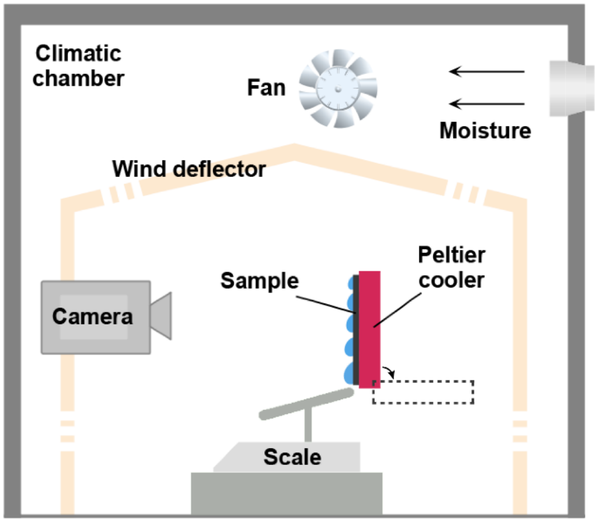

Figure S2. Schematic of the setup in climatic chamber, Related to Figure 1. The air temperature and relative humidity in chamber can be precisely controlled. The Peltier cooler was set either vertically or horizontally depending on the experiments.

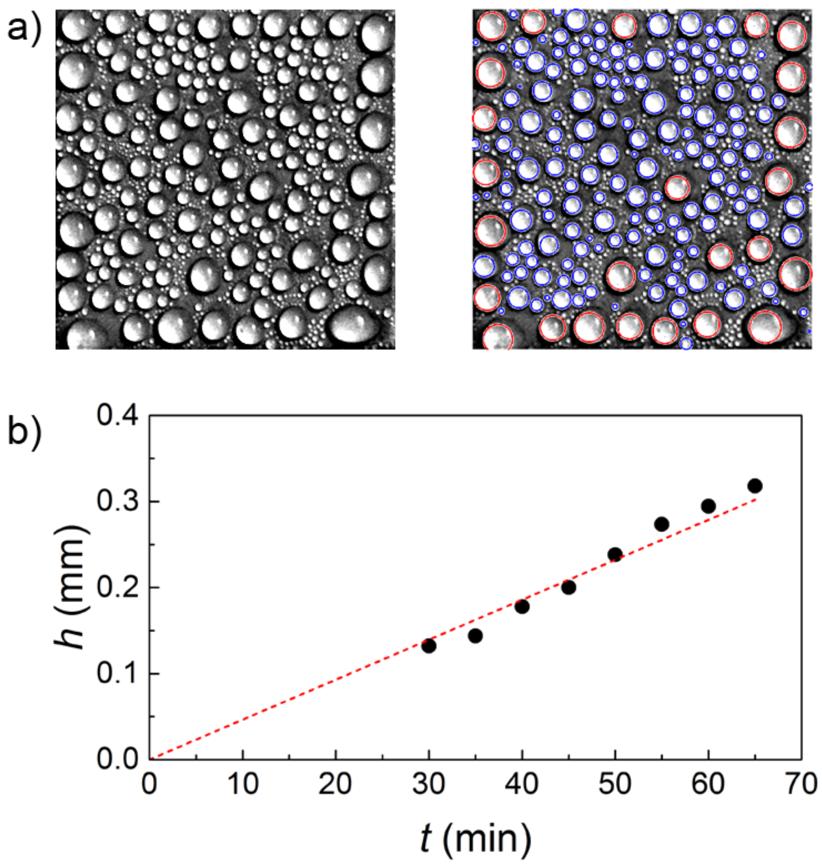

Figure S3. Condensation rate calculation, Related to Figure 1. a) Example of droplets recognition on tape surface (t=3600 s). b) The calculated equivalent water film thickness at different time during condensation. The dotted line represents the total volume of condensed water versus t, which is obtained by fitting the thickness data points.

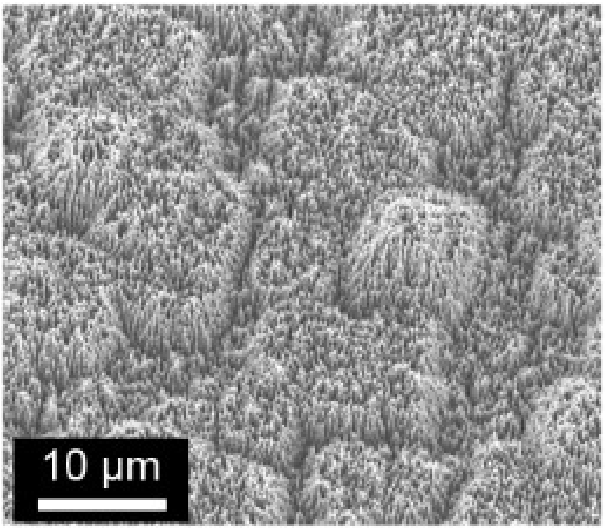

Figure S4. A SEM photo of *BS*, Related to Figure 1. The morphology of BS is similar to that of *BSP*, which signifies that the covered PTFE thin film will not change the surface morphology of Black Silicon.

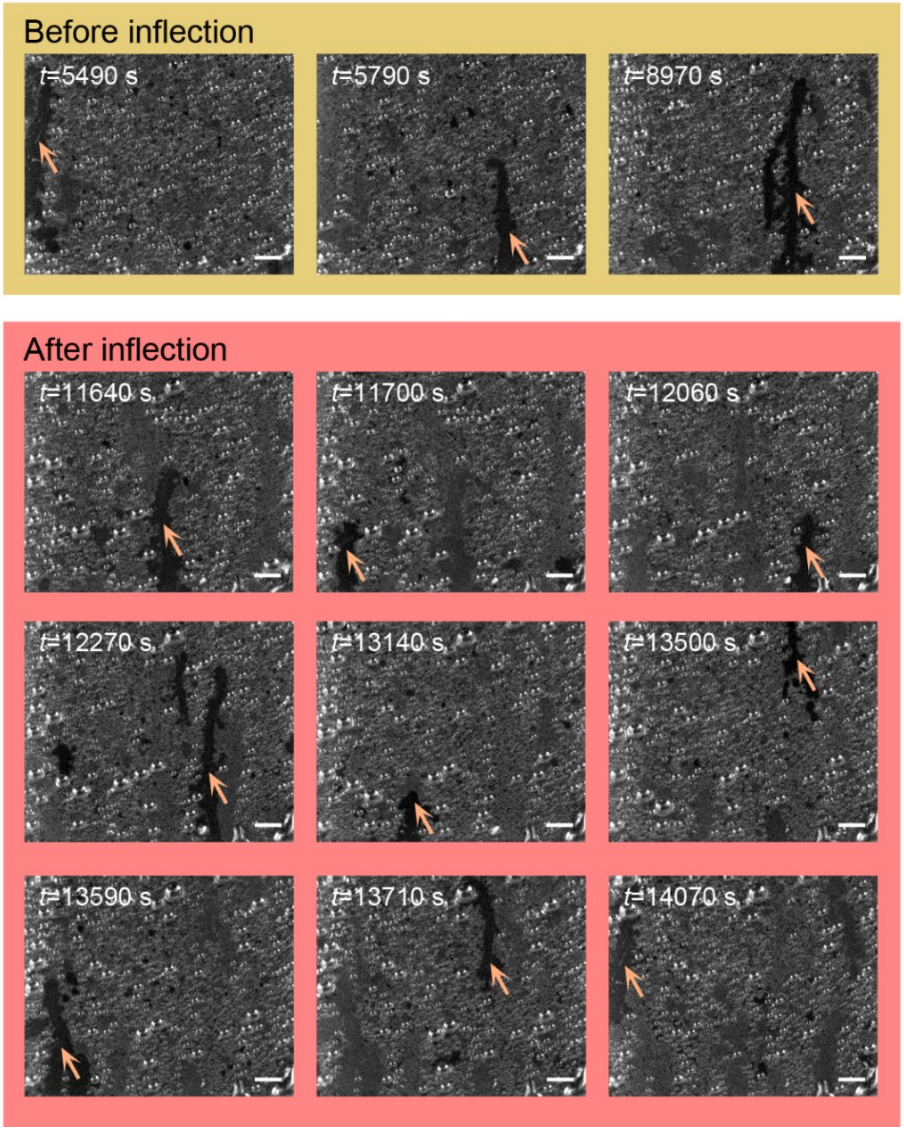

Figure S5. Sweeping events on vertical *BSP* surface during water harvesting experiment, Related to Figure 1-2. The remained traces after sweeping are marked by arrow symbols. The scale bars are 2 mm.

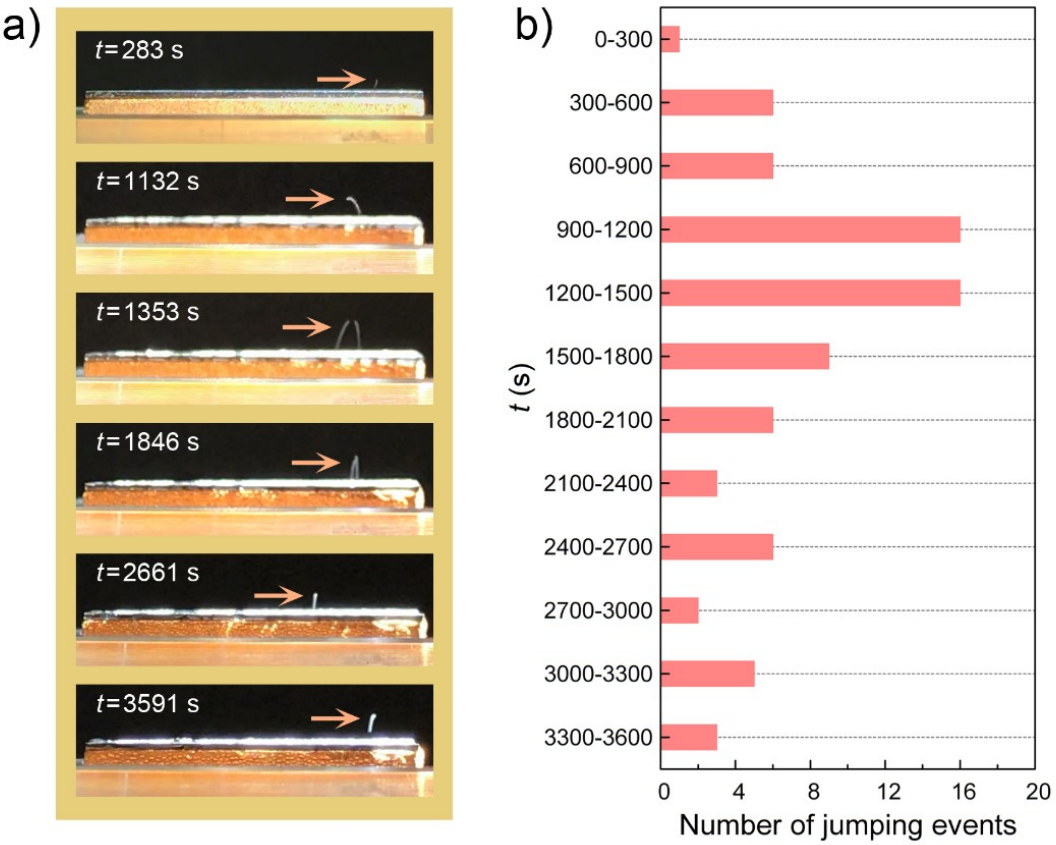

Figure S6. Jumping behavior observation on horizontal *BSP*, Related to Figure 3. a) Typical droplet jumping events in each time period. Parabolic trajectories are clearly visible. b) Statistics of jumping events in each time period. Jumping behaviors start from the early beginning period of condensation and appears throughout the whole condensation process, which is therefore able to drive the water removal from almost the beginning of condensation.

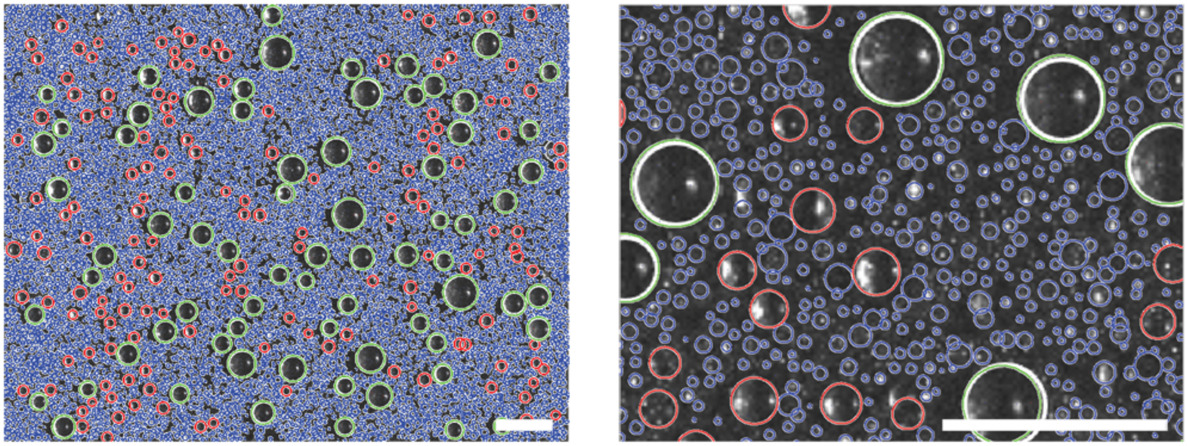

Figure S7. Typical droplets recognition on *BSP* (*t*=3600 s) , Related to Figure 4. Right picture shows the zoom-in details to intuitively exhibit the condensed droplet density. The scale bars in two pictures are 2 mm. The surface coverage on *BSP* can be then calculated by the statistical droplet number and the average droplet radius counted by Matlab. Notably, the calculated surface coverage is apparent coverage rather than the actual one.

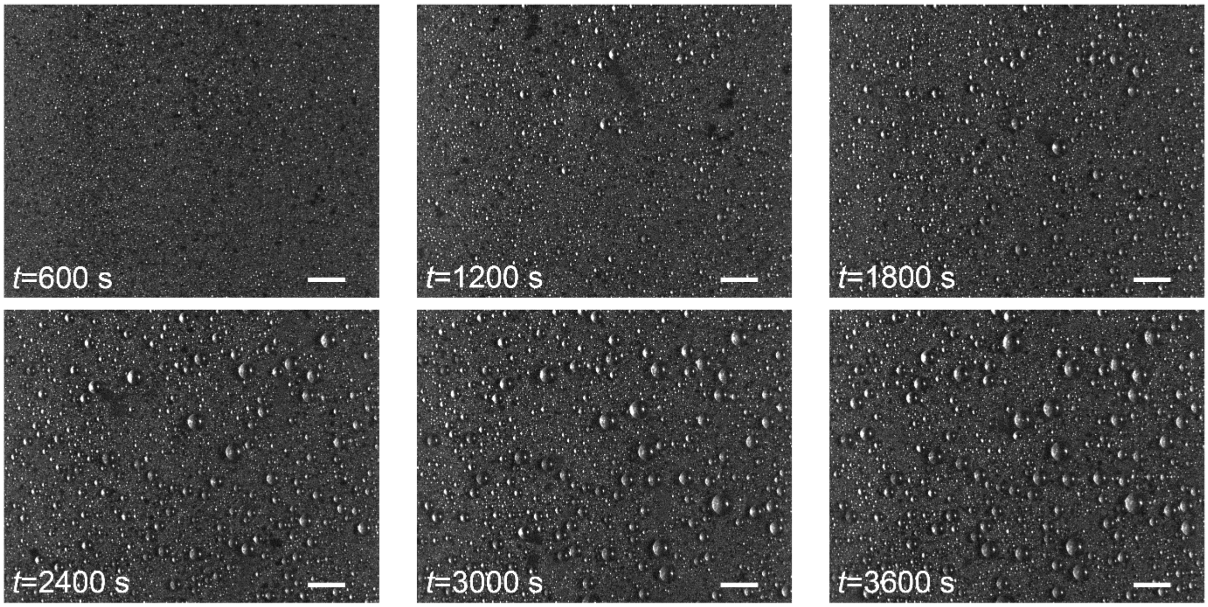

Figure S8. Top-view photos of droplets on horizontal *BSP* sample in each condensation period, Related to Figure 4. The scale bars are 2 mm. To reveal the droplet renewal evolution mode, the populations of droplet with different size have also been counted by Matlab.
